# Symptoms Based on Deficiency Syndrome in Traditional Chinese Medicine Might Be Predictor of Frailty in Elderly Community Dwellers

**DOI:** 10.1155/2021/9918811

**Published:** 2021-08-26

**Authors:** Zhen Qi, Bei-Ling Wu, Chuan Chen, Zhi-Hua Yu, Ding-Zhu Shen, Jiu-Lin Chen, Hong-Bin Zhao, Lin Sun

**Affiliations:** Shanghai Geriatric Institute of Chinese Medicine, Shanghai University of Traditional Chinese Medicine, 365 South Xiangyang Road, Shanghai 200031, China

## Abstract

**Background:**

The most widely used frailty phenotype and frailty indexes are either time-consuming or complicated, thus restricting their generalization in clinical practice; and therefore, an easier and faster screening tool is needed to be developed.

**Objective:**

To select sensitive symptoms in traditional Chinese medicine (TCM) and study whether they can improve the risk prediction of frailty.

**Methods:**

This is a cross-sectional study enrolling 2249 Chinese elderly community dwellers. Data were collected via face-to-face inquiries, anthropometric measurements, laboratory tests, and community health files. Frailty was the main outcome measure, and it was evaluated by Fried's frailty phenotype (FP). The ordinal logistic regression model was used to identify the factors associated with frailty. The risk assessment plot was used to compare the discriminative ability for frailty among models with and without TCM symptoms.

**Results:**

The identified sensitive influential factors for frailty included age, education level, dietary habits, chronic obstructive pulmonary disease, diabetes, cerebral infarction, osteoporosis, cold limbs, lethargy and laziness in speaking and moving, weakness of lower limbs, slow movement, dry mouth and throat, and glazed expression. The risk prediction for “frailty cumulative components ≥1” was not significantly increased, while for “frailty cumulative components ≥2”, a new model developed with the above selected TCM symptoms had a higher AUC than the baseline model without it (0.79 VS 0.81, *P*=0.002). And the NRI and IDI for the new model were 41.4% (*P*=0.016) and 0.024% (*P*=0.041), respectively.

**Conclusion:**

This research might provide an easier and faster way for early identification and risk prediction of frailty in elderly community dwellers.

## 1. Introduction

Frailty, characterized by an increased vulnerability to stressors due to a reduction in function across multiple physiological systems [1], is an emerging global health burden and gaining more and more international attention [2]. In the context of rapid growth in global aging population, especially in China, according to the latest systematic review that included 14 studies with total 81258 participants, the pooled prevalence of frailty and prefrailty among Chinese community-dwelling elderly has reached 10% (95% CI: 8% to 12%) and 43% (95% CI: 37% to 50%), respectively [3].

Frailty can lead to various adverse outcomes such as increased mortality, disability, falls, fractures, hospitalization and nursing home admission [4, 5]. Besides, it is also an important factor of disease progression and prognosis [6, 7]. Hence, alongside the prevalent frailty, frailty places a burden not only on affected individuals, their families, and caregivers but also on health and social care systems by increasing its related healthcare expenditure and use [8].

Since the landmark attempt was made to standardize the definition of frailty through the frailty phenotype by Fried in 2001 [9], many other frailty measurements are developed, such as the frailty indexes, the Clinical Frailty Scale (CFS), the Groningen Frailty Indicator, the Edmonton Frail Scale, and the FRAIL scale. Among the above-mentioned instruments, the most widely used in clinical practice are the frailty phenotype and frailty indexes, which are well validated in many populations and settings [10]. However, the main challenges for these two tools are that they are too complicated and time-consuming for use in clinical settings, which preclude their application to large-scale screens and routine physical examinations. Hence, they are applied for only a subset of patients, with most of the older people in a community or hospital not having their frailty assessed at all.

Previous studies demonstrated that physical or somatic symptoms might be associated with frailty [11]. However, it is not yet clear which symptoms act as the key role in the risk prediction of frailty. In traditional Chinese medicine (TCM), great importance is attached to symptoms because they are one of the important bases of syndrome differentiation and treatment. The onset characteristics and clinical manifestations of decreased physiological reserve function in frailty are consistent with symptoms of deficiency syndrome in TCM. Just as the whole process of human growth and aging described in the classic Huangdi Canon of Internal Medicine, it is emphasized that a frail body occurs with the growth of age due to the deficiency of Yin, Yang, qi, blood, and essence in five viscera. For example, qi deficiency can lead to fatigue, weakness, shortness of breath, unwilling to speak, etc. Blood deficiency can cause pale complexion and lip color, insomnia, poor memory, blurred vision, etc. Yin deficiency can result in hot flashes, night sweats, dry mouth and throat, etc. Yang deficiency can bring cold limbs and intolerance of cold. Essence deficiency is manifested as premature aging, weakness of lower limb, slow movement, glazed expression, hair loss, loose teeth, etc.

Thus, it is necessary to better understand risk factors for the development of frailty based on the integrated Chinese and Western medicine, which facilitates optimal disease prevention and treatment strategies. However, it is not yet clear what are the core TCM symptoms based on deficiency syndrome that are associated with frailty. No study has investigated the role of TCM symptoms based on deficiency syndrome combined with traditional risk factors on the prediction of frailty in the community dwellers. The purpose of this study was to identify the core TCM symptoms based on deficiency syndrome associated with frailty and investigate whether they can improve the risk prediction of frailty beyond traditional frailty risk factors, which might provide an easier and faster way for early identification and risk prediction of frailty in elderly community dwellers.

## 2. Methods and Materials

### 2.1. Design and Study Population

This is a population-based cross-sectional study whose data come from the longitudinal study of frailty in Shanghai older people, which is registered in the website of Chinese Clinical Trial Registry (ChiCTR2000039491). And it is designed to be a simple random sampling survey, and therefore its smallest sample size is calculated according to the following formula:

(1) $$\begin{array}{c} n=\frac{u_{\alpha /2}^{2}\pi \left(1-\pi \right)}{\delta^{2}}. \end{array}$$

In the above formula, *π* refers to the prevalence rate, and *δ* is the allowable error; set *δ* = 0.1*π*, *α* = 0.05, and u_*α*/2_ = 1.96.

According to available domestic reports in China, the prevalence of frailty and prefrailty in the elderly aged over 65 was 10% and 43%, respectively. Therefore, the estimated results of the smallest sample size are as follows:

(2) $$\begin{array}{c} 1.96^{2}\times 10\%\times \frac{\left(1-10\%\right)}{{\left(0.1\times 10\%\right)}^{2}}=3458,\\ 1.96^{2}\times 43\%\times \frac{\left(1-43\%\right)}{{\left(0.1\times 43\%\right)}^{2}}=510. \end{array}$$

Considering the 20% failure rate of the cohort survey, the smallest sample size of subjects selected in this project should be between 612 and 4150. However, for the limitation of funding of this project, eventually, by using a simple random sampling method, we randomly selected 2500 subjects from the annual physical examination population of eight community centers in Shanghai during September 2018 to December 2019. Then, 2249 subjects were enrolled according to the following inclusion criteria: 1) age more than 65 according to the 2019 ICFSR international clinical practice guidelines for identification and management of frailty [12] and 2) complete data on Fried's frailty phenotype (FP) evaluation, general information (sex, BMI, waistline, hipline, marital status, education level, living status, living stairs, living area, dietary habit, exercise frequency and variety), history of chronic disease and medication, cognitive function, and the TCM symptoms based on deficiency syndrome. Subjects whose physical functions are affected by disabilities and fractures; subjects with serious mental disorders (schizophrenia, depression, anxiety); and subjects with dementia were excluded.

The Medical Ethics Committee of the Longhua Hospital affiliated with the Shanghai University of TCM approved the study, and written informed consent was obtained from all included subjects according to the Declaration of Helsinki.

### 2.2. Data Collection

Patients' metadata were collected (i.e., general information, history of chronic disease and medication, cognitive function, and the TCM symptoms based on deficiency syndrome) by community physicians. All participating community physicians were asked to receive 1–2 days of intensive training of research-related skills and techniques until they are qualified. Then, face-to-face inquiries between them and patients were conducted through electronic small programs on mobile phones we developed previously and the paper version of the form of questionnaire. The cognition of subjects was evaluated by clock drawing task (CDT) [13] and Mini-Mental State Examination (MMSE) [14].

### 2.3. Frailty Assessment

Fried's frailty phenotype was used in the frailty assessment [9], and the five aspects were defined as follows: ① unintentional weight loss: self-reported weight loss or measured weight loss of ≥5% in the past year; ② decreased grip strength: lowest quintile stratified by sex and body mass index (In males, decreased grip strength is defined as grip strength ≤29 kg, ≤30 kg, and ≤32 kg, respectively, for BMI ≤ 24 kg/m^2^, 24.1–28 kg/m^2^, and >28 kg/m^2^. In females, it is defined as grip strength ≤17 kg, ≤17.3 kg, ≤18 kg, and ≤21 kg, respectively, for BMI ≤ 23 kg/m^2^, 23.1–26 kg/m^2^, 26.1–29 kg/m^2^, and >29 kg/m^2^); ③ slow gait speed: lowest quintile of gait speed (m per s) stratified by sex and height (4.57 m walk time ≥7 s and ≥6 s for males with height of ≤173 cm and >173 cm, respectively; 4.57 m walk time ≥7 s and ≥6 s for females with height of ≤159 cm and >159 cm, respectively); ④ low physical activity: low energy expenditure, based on physical activity questionnaire; and ⑤ self-reported exhaustion. Stratification of frailty was defined as nonfrail (0 criteria present), prefrail (1–2 criteria present), and frail (≥3 criteria present).

### 2.4. TCM Symptoms Based on Deficiency Syndrome

There is no specific available reference standard for the syndrome differentiation for deficiency syndrome in the elderly. Therefore, in our previous research, on the main basis of the available criteria (Clinic Terminology of Traditional Chinese Medical Diagnosis and Treatment—Syndromes) published by the Chinese National Administration of TCM [15], we constructed a framework of symptoms for deficiency syndrome in the elderly. Then, by inviting 31 TCM gerontologists in China and carrying out the Delphi research [16], TCM symptoms based on deficiency syndrome, which consist of 63 items, were finally collected (see Table 1). At last, a questionnaire of TCM symptoms based on deficiency syndrome was formed to confirm the presence of these symptoms among community elders.

### 2.5. Data Analysis

If not otherwise stated, data are presented with mean ± SD or proportions (in percentages). In the continuous data, ANOVA multiple comparison tests or independent sample Kruskal–Wallis H (*k*) tests were used to compare differences between the three groups (nonfrailty, prefrailty, and frailty groups) for normally or non-normally distributed data, respectively. In the categorical data, Pearson's x^2^ test was used, and the Bonferroni correction was used to adjust the *P* value for the multiple comparisons.

To identify the possible risk factors associated with frailty progression, ordinal regression analysis was performed in the premise of satisfying the test of parallel lines.

To compare the discriminative ability among models with and without TCM symptoms based on deficiency syndrome for the risk prediction of frailty, the risk assessment plot was used [17]. The baseline model included only the traditional identified risk factors associated with frailty progression, whereas the new model included the baseline model and TCM symptoms based on deficiency syndrome. Then, the area under the receiver-operating characteristic curve (AUC), net reclassification improvement (NRI), and integrated discrimination improvement (IDI) were calculated. The area under the receiver-operating characteristic curve was a summary measure for discrimination between individuals developing specific frailty cumulative components and those who did not. Net reclassification improvement focuses on reclassification tables constructed separately for participants with and without events and quantifies the correct movement in categories—upward for events and downward for nonevents when adding the new risk factor [17]. Integrated discrimination improvement measures how the *R*^2^ (explained variance) improves with the addition of the new risk factor [18]. Because no established net reclassification improvement categories exist to guide clinical decisions for frailty risk in Chinese adults, we only calculated continuous net reclassification improvement.

All statistical analyses were performed with IBM spss21.0 (IBM Corporation, Armonk, NY, USA), and the risk assessment plot was conducted in Matlab R2016a. All reported *P* values were 2-tailed, and those <0.05 were considered statistically significant.

## 3. Results

### 3.1. Baseline Characteristics of the Study Subjects

The baseline characteristics of 2249 participants are shown in Table 2. In the study cohort, 739 and 55 subjects met the prefrailty and frailty criteria, respectively, whereas 1455 subjects were in the nonfrailty group. The results suggest that along with the change of the frailty stage from nonfrail to prefrail and then to frail, age, waist line, the percentages of being widowed, low educational level (primary school and illiterate or semi-illiterate), living alone or in nursing home, living on second floor or above without elevator, vegan dietary habit, and none or very little exercise are all gradually increased (*P* < 0.05), while the percentage of exercise diversity (at least 2 types of exercise) is gradually decreased (*P* < 0.05). Moreover, compared with the nonfrailty group and prefrailty group, the participants of the frailty group had a higher WHR and percentage of living in suburban (*P* < 0.05).

### 3.2. Past Medical History and Cognition Function of the Study Subjects

Past medical history and cognition function of 2249 participants for studied three frailty stages are shown in Table 3. The results show that a gradual higher number of the cumulative chronic diseases occurred in the prefrail group and frail group compared with the nonfrail group (*P* < 0.05). Furthermore, among all the studied chronic diseases in our research, we found that the proportions of cerebral infarction, chronic heart disease (CHD), diabetes, osteoporosis, and chronic obstructive pulmonary disease (COPD) were gradually increased alongside the aggravation of frailty stages (*P* < 0.05). Meanwhile, more cumulative medication types of the prefrail group and frail group are observed compared with nonfrail group (*P* < 0.05). As for the cognition function of three studied groups, the result manifests that the frail group had a declined cognition function compared with the nonfrail group and prefrail group, which indicated by the reduced total scores of both clock drawing test (CDT) and Mini-Mental State Examination (MMSE) (*P* < 0.05).

### 3.3. TCM Symptoms Based on Deficiency Syndrome of the Study Subjects

Distribution of TCM symptoms based on deficiency syndrome of 2249 participants for three studied frailty stages is shown in Table 4. Here, among all 63 TCM symptoms based on deficiency syndrome, we only showed 28 of those having a statistical significance for their proportion in three studied frailty stages (*P* < 0.05). The proportion of these 28 symptoms occurred in the frailty group from high to low is as follows: tinnitus or deafness, dizziness, cold limbs, numbness in the hand and feet, fear of wind and coldness, rare and degenerate eyebrows, lethargy and laziness in speaking and moving, fatigue, chest distress, soreness of four limbs, weakness of lower limbs, dry throat and mouth, frequent urination or nocturnal enuresis more than 3 times, short of breath, susceptibility to common cold, white and clear phlegm, feverishness in palms and soles, slow movement, urinary incontinence or dribbling urination, dyspnea and tachypnea during activity, constipation, less sticky or bloody phlegm, night sweating, abdominal pain relived by warmness and pressure, faint low voice, spontaneous perspiration, a trapped heavy body, and glazed expression.

### 3.4. Identified Risk Factors Associated with Frailty Cumulative Components

By ordinal regression analysis, the identified risk factors associated with frailty cumulative components are shown in Table 5. The results demonstrated that the following factors influence frailty progression: age (OR = 1.05, *P* < 0.001), educational level (taking illiterate or semi-illiterate as the reference, the OR values of university diploma and above, high school/technical secondary school, junior high school, and primary school are 0.60, 0.74, 0.74, and 0.78, respectively, *P* < 0.05), dietary habit (taking vegan as a reference, the OR values of prefer to meat, prefer to vegetables, and balanced diet were 0.47, 0.38, and 0.39, respectively, *P* < 0.05), osteoporosis (the OR value of absence of this disease is 0.80, *P*=0.036), chronic obstructive pulmonary disease (the OR value of absence of this disease is 0.64, *P*=0.025), cerebral infarction (the OR value of absence of this disease is 0.76, *P*=0.002), diabetes (the OR value of absence of this disease is 0.85, *P*=0.043), cold limbs (the OR value of absence of this symptom is 0.86, *P*=0.023), lethargy and laziness in speaking and moving (the OR value of absence of this symptom is 0.85, *P*=0.039), weakness of lower limbs (the OR value of absence of this symptom is 0.77, *P*=0.001), dry mouth and throat (the OR value of absence of this symptom is 0.87, *P*=0.031), slow movement (the OR value of absence of this symptom is 0.63, *P* < 0.001), and glazed expression (the OR value of absence of this symptom is 0.57, *P*=0.015).

### 3.5. Comparison of Models with/without Selected TCM Symptoms for the Prediction of Frailty

**Figure 1:**
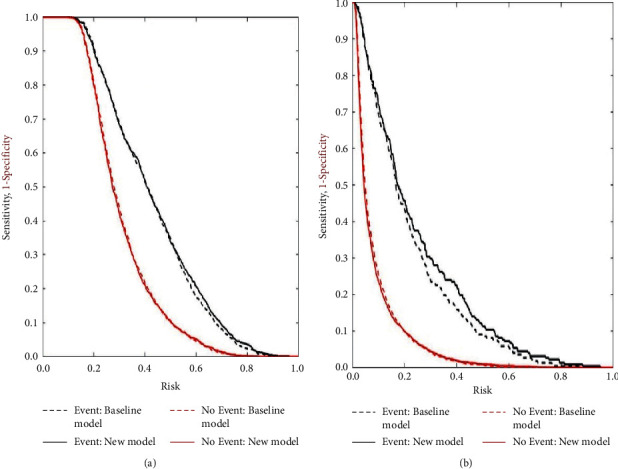
Additional value of TCM symptoms based on deficiency syndrome compared with the baseline model for the prediction of frailty. Risk assessment plots for the baseline model (dashed lines) and new model including selected TCM symptoms based on deficiency syndrome (solid lines). Event curves (black lines) represent sensitivity vs calculated risk. No event curves (red lines) represent 1 − specificity vs calculated risk. Baseline model: age, education level, dietary habits, chronic obstructive pulmonary disease, diabetes, cerebral infarction, osteoporosis. New model: baseline model + selected TCM symptoms based on deficiency syndrome (cold limbs, lethargy and laziness in speaking and moving, weakness of lower limbs, slow movement, dry mouth and throat, glazed expression). The left figure (a) shows the prediction for frailty cumulative components “≥1”, and the right figure (b) shows the prediction for frailty cumulative components “≥2”.

Two models with/without selected TCM symptoms based on deficiency syndrome was compared for their ability to classify participants into the group with more than specific frailty cumulative components and the group without (Table 6). Here, considering the small sample size of the frailty group (frailty cumulative components ≥3), we only observed the additional value of related TCM symptoms for the risk prediction of frailty cumulative components “≥1” and “≥2”. The baseline model included age, education level, dietary habits, chronic obstructive pulmonary disease, diabetes, cerebral infarction, and osteoporosis. In the ordinal regression analysis, in addition to the above traditional risk factors, TCM symptoms based on deficiency syndrome (cold limbs, lethargy and laziness in speaking and moving, weakness of lower limbs, slow movement, dry mouth and throat, glazed expression) were also associated with frailty progression. Therefore, the new model additionally included above 6 selected TCM symptoms based on deficiency syndrome. The risk prediction for “frailty cumulative components ≥1” was not significantly increased, while for “frailty cumulative components ≥2”, the new model with above selected TCM symptoms had a higher AUC (0.81) than the baseline model without it (0.79, *P*=0.002). And the NRI and IDI for the new model were 41.4% (*P*=0.016) and 0.024% (*P*=0.041), respectively. The risk assessment plot supported the additional value of TCM symptoms based on deficiency syndrome in the incident frailty risk assessment (Figure 1).

## 4. Discussion

The present study examined the effects of TCM symptoms based on deficiency syndrome on the risk prediction of frailty among Chinese community dwellers and produced 2 main findings. First, in addition to traditional frailty risk factors (age, education level, dietary habits, chronic obstructive pulmonary disease, diabetes, cerebral infarction, osteoporosis), there were 6 core TCM symptoms based on deficiency syndrome (cold limbs, lethargy and laziness in speaking and moving, weakness of lower limbs, slow movement, dry mouth and throat, glazed expression), which directly and independently associated with frailty progression. Second, these 6 TCM symptoms based on deficiency syndrome improved the incident frailty risk prediction beyond the above-mentioned traditional frailty risk factors.

The findings show frailty is associated with age, lower educational level, poor diet, and certain chronic diseases (chronic obstructive pulmonary disease, diabetes, cerebral infarction, osteoporosis), which is almost consistent with the available literature report. For example, a longitudinal study from 13 years' follow-up of 1205 older adults demonstrated that older adults with a low educational level had higher odds of being frail compared with those with a high educational level (relative index of inequality odds ratio, 2.94), and 76% of the impact of educational level on frailty was related to income, self-efficacy, cognitive impairment, obesity, and the number of chronic diseases [19]. Besides, poor diet quality increases the risk of frailty and its consequences by resulting in malnutrition [20], and it is recommended that the promotion of a Mediterranean diet and a protein intake of at least 1–1.2 g per kilogram of body weight per day is beneficial for frail patients, and vitamin D supplementation is needed for those who are at an elevated risk of falls and fractures [21]. As for the correlation of frailty with chronic diseases, a large-scale study that enrolled 493737 participants aged 37–73 years with a median of 7 years' follow-up show that chronic obstructive pulmonary disease (5.6; 95% CI: 5.2–6.1) and diabetes (5.0; 95% CI: 4.7–5.2) are among the top five long-term conditions associated with frailty. However, other frailty-related long-term conditions have also been mentioned, such as multiple sclerosis, chronic fatigue syndrome, connective tissue disease, and coronary heart disease [22]. Even if there are some differences due to some inclusion bias and different sample size, our outcomes of the association between chronic disease condition and frailty were substantially in line with the above related reports; further studies could be carried out to include other broader chronic diseases and larger sample size in the future.

By identifying the 6 TCM symptoms based on the deficiency (cold limbs, lethargy and laziness in speaking and moving, weakness of lower limbs, dry mouth and throat, slow movement, and glazed expression) as the key influential factors for frailty progression, we also investigated their role in the risk prediction of frailty. We have shown for the first time that these 6 TCM symptoms, which were significantly more prevalent in prefrail and frail patients and associated with frailty scores, are independent additive predictors of incident frailty beyond the traditional frailty risk factors. As these symptoms are all selected from the deficiency syndrome, therefore, according to the TCM theory, weakness of lower limbs, slow movement, and glazed expression can be attributed to the kidney-essence deficiency; cold limbs is a typical manifestation of Yang deficiency; dry mouth and throat are typical hints of Yin deficiency; and the symptom of lethargy and laziness in speaking and moving is mainly a suggestion of qi deficiency. In summary, the main TCM pathogenesis of frailty is kidney-essence deficiency, accompanied with qi deficiency, Yin deficiency, and Yang deficiency, which would provide a foundation for the TCM treatment in frailty.

Among the above-mentioned 6 TCM symptoms based on the deficiency, similar phenomena like weakness of lower limbs and slow movement were also reported in other studies to be related with frailty occurrence. For example, de Amorim [23] once reported that poor lower-limb performance was one of the factors associated with frailty. Furthermore, some wireless sensor technologies like an eChair were used to detect slowness in movement, weakness, and weight loss so as to assess frailty [24]. Besides, another study investigated the role of daily electromyography (EMG) recordings of muscle activity in dissociating stages of frailty in females with Parkinson's disease (PD), and they found that slower movement was caused by longer burst durations, which suggests that more muscle activity is required to initiate movement [25]. Another frailty-related TCM symptom glazed expression may be an external manifestation description of dementia, which may increase prevalence of frailty [26].

In our research, we also found TCM symptoms like cold limbs can predict frailty incidence, this symptom was caused by more sensitive perception of cold, which was firstly reported in diabetic patients with polyneuropathy lesions [27], and it is observed that an elevated cold perception thresholds for the foot was the most pronounced sensory defect in patients with type 1 diabetes mellitus [28]. As we mentioned above, diabetes is an important risk factor related to frailty; therefore, further research is needed to determine which body part of frail elders is most sensitive to cold and the causal relationship among weakness, diabetes, and cold limbs.

Another frailty-related TCM symptom we identified is dry throat and mouth, namely oral dryness or xerostomia, which is a common phenomenon in the older population. Oral dryness can be caused by many reasons, such as certain drugs, diabetes, head and neck radiotherapy, and systemic diseases like various connective tissue disorders, but the most common cause is the use of xerogenic drugs, for example, diuretics, antidepressants, neuroleptics, cytostatics, antiparkinsonism drugs, antihypertensives, and antihistaminics [29]. In addition, the reported prevalence of xerostomia in older population is lower in men (10–26%) than in women (10–33%). Moreover, it is observed that menopausal women with oral dryness feeling had higher serum and salivary testosterone and lower femur BMD [30]. As for the mechanisms of oral dryness, a reduction in the secretion of some antimicrobial substances like lactoferrin and chromogranin A may be associated with oral dryness [31].

To the best of our knowledge, this is the first retrospective study to show that TCM symptoms based on deficiency syndrome are significant predictors of frailty. This research might provide an easier and faster way for early identification and risk prediction of frailty in elderly community dwellers. Our study has some limitations. A major limitation of the present study is that this is a cross-sectional study and we only found that TCM symptoms based on deficiency syndrome might improve the incident frailty risk prediction beyond the traditional risk factors. Further longitudinal studies should be conducted to determine whether these symptoms evolve in the clinical development of the frailty. Second, in this retrospective study, we only investigated the TCM symptoms based on deficiency syndromes. However, TCM emphasizes the combined use of the four diagnostic methods in syndrome differentiation; further study that involves tongue manifestations and pulse conditions additionally should be carried out in the future. Third, this is a single-center study, which only involves Shanghai's community elders, and therefore, a multicenter research should be further carried out all over China to verify this result.

## 5. Conclusion

Our current findings suggest that the 6 TCM symptoms based on deficiency syndrome (cold limbs, lethargy and laziness in speaking and moving, weakness of lower limbs, slow movement, dry mouth and throat, glazed expression) are directly associated with frailty progression. And compared with the baseline model which only involves conventional factors, the new model additionally added with the above-mentioned 6 selected TCM symptoms can significantly improve the risk prediction for “frailty cumulative components ≥2”. Therefore, screening of these 6 TCM symptoms might provide an easier and faster way for early identification and risk prediction of frailty in elderly community dwellers.

## Figures and Tables

**Table 1:** List of 63 TCM symptoms based on deficiency syndrome.

01. Dizziness	22. Abdominal pain relived by warmness and pressure	43. Insomnia
02. Forgetfulness	23. Sore pain in loin and legs	44. Dreaminess during sleep
03. Tinnitus or deafness	24. Cramp in the heel-to-shin area	45. Easy to sleep after excessive sitting during the day
04. Blurred vision	25. Weakness of lower limbs	46. Early awakening
05. Dryness in the eyes	26. Tremble or wriggle limbs	47. Frequent urination or nocturnal enuresis more than 3 times
06. Dry throat and mouth	27. Soreness of four limbs	48. Urinary incontinence or dribbling urination
07. Saliva flowing out of mouth	28. Numbness in the hand and feet	49. Thin sloppy stool
08. Faint low voice	29. Kraurotic fingernail	50. Dawn diarrhea
09. Hoarseness	30. Fear of wind and coldness	51. Constipation
10. Palpitation	31. Cold limbs	52. Emaciation
11. Chest distress	32. Feverishness in palms and soles	53. Dry or itchy skin
12. Chest pain	33. Hectic fever	54. Edema of lower limbs
13. Short of breath	34. Night sweating	55. A trapped heavy body
14. Dyspnea and tachypnea during activity	35. Spontaneous perspiration	56. Hair loss and loose teeth
15. Repeated cough	36. Susceptibility to common cold	57. Pale complexion and lip color
16. Repeated dry cough	37. Fatigue	58. Flushing of both cheekbones
17. White and clear phlegm	38. Lethargy and laziness in speaking and moving	59. Pale and lackluster face
18. Less sticky or bloody phlegm	39. Slow movement	60. Sallow complexion
19. Decreased appetite	40. Glazed expression	61. Pallid complexion
20. Pressure-relieved abdominal distension	41. Intranquil feeling caused by being frightened or vigilant	62. Rare and degenerate eyebrows
21. Bearing-down distension of abdomen	42. Restless	63. Emotional sadness

**Table 2:** The basic characteristics of the study subjects.

Variables	Frailty stage			*P* value
	Nonfrailty group	Prefrailty group	Frailty group	
	(*n* = 1455)	(*n* = 739)	(*n* = 55)	
Basic information				
Age	70 [67–74]	72 [69–77]	78 [70–82]	＜0.001
Sex (male)	677 [46.5%]	340 [46.0%]	18 [32.7%]	0.131
BMI	24.54 [22.58–26.56]	24.38 [22.03–26.67]	23.67 [21.84–27.63]	0.178
Waist line	84.00 [78.00–90.00]	85.00 [78.00–90.20]	88.50 [80.00–93.00]	0.008
Hip line	94.00 [89.78–98.40]	95.00 [90.00–100.00]	95.65 [90.03–102.00]	0.194
WHR	0.89 [0.86–0.93]	0.89 [0.86–0.93]	0.91 [0.87–0.97]	0.027

Marital status				
Married	1326 [91.1%]	623 [84.3%]	42 [76.4%]	＜0.001
Unmarried	2 [0.1%]	6 [0.8%]	0 [0.0%]	
Divorced	6 [0.4%]	6 [0.8%]	0 [0.0%]	
Widowed	121 [8.3%]	104 [14.1%]	13 [23.6%]	

Educational level				
University diploma or above	158 [10.9%]	62 [8.4%]	4 [7.3%]	＜0.001
High school/technical school	267 [18.4%]	136 [18.4%]	4 [7.3%]	
Junior high school	493 [33.9%]	216 [29.2%]	8 [14.5%]	
Primary school	317 [21.8%]	164 [22.2%]	13 [23.6%]	
Illiterate or semi-illiterate	220 [15.1%]	161 [21.8%]	26 [47.3%]	

Living status				
Living with spouse/children	1337 [91.9%]	641 [86.7%]	43 [78.2%]	＜0.001
Living alone	117 [8.0%]	92 [12.4%]	9 [16.4%]	
Living in nursing home	1 [0.1%]	6 [0.8%]	3 [5.5%]	

Residential floor				
1/F	267 [18.4%]	186 [25.2%]	17 [30.9%]	0.001
≥2/F with elevator	187 [12.9%]	98 [13.3%]	8 [14.5%]	
≥2/F without elevator	1001 [68.8%]	455 [61.6%]	30 [54.5%]	
Residential area (suburban)	575 [39.5%]	274 [37.1%]	31 [56.4%]	0.016

Dietary habit				
Prefer to meat	67 [4.6%]	40 [5.4%]	3 [5.5%]	
Balanced diet	752 [51.7%]	388 [52.5%]	19 [34.5%]	0.002
Prefer to vegetables	635 [43.6%]	305 [41.3%]	31 [56.4%]	
Vegan	1 [0.1%]	6 [0.8%]	2 [3.6%]	

Exercise frequency				
≥1 time/day	870 [59.8%]	419 [56.7%]	14 [25.5%]	
≥1 time/week	151 [10.4%]	79 [10.7%]	10 [18.2%]	＜0.001
None or very little	434 [29.8%]	241 [32.6%]	31 [56.4%]	

Exercise types				
0	357 [24.5%]	188 [25.4%]	27 [49.1%]	
1	1031 [70.9%]	531 [71.9%]	28 [50.9%]	0.001
2	63 [4.3%]	17 [2.3%]	0 [0.0%]	
3	4 [0.3%]	3 [0.4%]	0 [0.0%]	

Smoking				
No	1048 [72.0%]	545 [73.7%]	47 [85.5%]	
Ever drinking	169 [11.6%]	78 [10.6%]	5 [9.1%]	0.190
Current smoking	238 [16.4%]	116 [15.7%]	3 [5.5%]	

Drinking				
No	1090 [74.9%]	578 [78.2%]	50 [90.9%]	
Ever drinking	91 [6.3%]	45 [6.1%]	2 [3.6%]	0.056
Little drinking	270 [18.6%]	115 [15.6%]	3 [5.5%]	
Excessive drinking	4 [0.3%]	1 [0.1%]	0 [0.0%]	

Data shown are median (interquartile range) or proportions (percentage). WHR: waist-to-hip ratio; 1/F: the first floor; 2/F: the second floor.

**Table 3:** Past medical history and cognition function of the study subjects.

Variables	Frailty stage			*P* value
	Nonfrailty group	Prefrailty group	Frailty group	
	(*n* = 1455)	(*n* = 739)	(*n* = 55)	
Chronic disease history				
The cumulative chronic diseases	1 [0–2]	1 [1–2]	2 [1–3]	＜0.001
Hypertension	777 [53.4%]	429 [58.1%]	35 [63.6%]	0.052
Cerebral infarction	149 [10.2%]	128 [17.3%]	25 [45.5%]	＜0.001
CHD	195 [13.4%]	146 [19.8%]	17 [30.9%]	＜0.001
Diabetes	237 [16.3%]	167 [22.6%]	16 [29.1%]	＜0.001
Osteoporosis	89 [6.1%]	74 [10.0%]	10 [18.2%]	＜0.001
Hyperlipemia	171 [11.8%]	99 [13.4%]	6 [10.9%]	0.515
COPD	17 [1.2%]	22 [3.0%]	4 [7.3%]	0.001
Chronic liver disease	16 [1.1%]	8 [1.1%]	3 [5.5%]	0.050
Advanced tumor	40 [2.7%]	16 [2.2%]	3 [5.5%]	0.357
Gout	38 [2.6%]	31 [4.2%]	1 [1.8%]	0.121
Pneumonia	9 [0.6%]	6 [0.8%]	1 [1.8%]	0.290
Cerebral hemorrhage	6 [0.4%]	6 [0.8%]	0 [0.0%]	0.432
Parkinson's disease	6 [0.4%]	7 [0.9%]	0 [0.0%]	0.323
Chronic nephrosis	22 [1.5%]	8 [1.1%]	1 [1.8%]	0.494
Cumulative medication type	0 [0–1]	1 [0–2]	1 [0–2]	＜0.001

Cognition function				
Total score of CDT	4 [3–4]	4 [2–4]	2 [1–4]	＜0.001
Total score of MMSE	29 [26–30]	29 [25–32]	26 [17–32]	0.007

Data shown are median (interquartile range) or proportions (percentage). CHD: chronic heart disease; COPD: chronic obstructive pulmonary disease; CDT: clock drawing test; MMSE: Mini–Mental State Examination.

**Table 4:** Distribution of TCM symptoms based on deficiency syndrome of different frailty stages.

No.	Items	Frailty stages			*P* value
		Nonfrailty group	Prefrailty group	Frailty group	
		(*n* = 1455)	(*n* = 739)	(*n* = 55)	
03	Tinnitus or deafness	596 [41.0]	322 [43.7]	34 [61.8]	0.006
01	Dizziness	474 [32.6]	296 [40.2]	28 [50.9]	＜0.001
31	Cold limbs	433 [29.8]	296 [40.2]	27 [49.1]	＜0.001
28	Numbness in the hand and feet	424 [29.1]	191 [25.9]	24 [43.6]	0.012
30	Fear of wind and coldness	427 [29.3]	272 [37.0]	23 [41.8]	＜0.001
62	Rare and degenerate eyebrows	378 [26.0]	233 [31.6]	23 [41.8]	0.002
38	Lethargy and laziness in speaking and moving	225 [15.5]	200 [27.2]	23 [41.8]	＜0.001
37	Fatigue	317 [21.8]	212 [28.8]	22 [40.0]	＜0.001
11	Chest distress	340 [23.4]	195 [26.5]	22 [40.0]	0.009
27	Soreness of four limbs	271 [18.6]	150 [20.4]	22 [40.0]	＜0.001
25	Weakness of lower limbs	210 [14.4]	194 [26.3]	22 [40.0]	＜0.001
06	Dry throat and mouth	428 [29.4]	253 [34.3]	20 [36.4]	0.045
47	Frequent urination or nocturnal enuresis more than 3 times	301 [20.7]	201 [27.3]	19 [34.5]	＜0.001
13	Short of breath	254 [17.5]	154 [20.9]	15 [27.8]	0.035
36	Susceptibility to common cold	189 [13.0]	122 [16.6]	15 [27.3]	0.002
17	White and clear phlegm	190 [13.1]	111 [15.1]	15 [27.3]	0.008
32	Feverishness in palms and soles	140 [9.6]	81 [11.0]	14 [25.5]	0.001
39	Slow movement	92 [6.3]	115 [15.6]	14 [25.5]	＜0.001
48	Urinary incontinence or dribbling urination	146 [10.0]	103 [14.0]	12 [21.8]	0.001
14	Dyspnea and tachypnea during activity	174 [12.0]	125 [17.0]	12 [21.8]	0.001
51	Constipation	148 [10.2]	68 [9.2]	12 [21.8]	0.012
18	Less sticky or bloody phlegm	118 [8.1]	80 [10.9]	12 [21.8]	0.001
34	Night sweating	152 [10.4]	112 [15.2]	11 [20.0]	0.001
22	Abdominal pain relived by warmness and pressure	75 [5.2]	59 [8.0]	8 [14.5]	0.003
8	Faint low voice	41 [2.8]	34 [4.6]	8 [14.5]	＜0.001
35	Spontaneous perspiration	160 [11.0]	115 [15.6]	7 [12.7]	0.009
55	A trapped heavy body	108 [7.4]	86 [11.7]	7 [12.7]	0.003
40	Glazed expression	10 [0.7]	11 [1.5]	7 [12.7]	＜0.001

Data are shown in proportions [percentages].

**Table 5:** Risk factors associated with frailty progression.

Variables entered	*β*	Se	Wald *χ*^2^ value	OR (95% CI)	*P* value
Age	0.05	0.01	72.9	1.05 [1.04–1.06]	＜0.001

Educational level					
University diploma or above	−0.51	0.13	16.43	0.60 [0.47–0.77]	＜0.001
High school/technical school	−0.30	0.11	7.76	0.74 [0.60–0.91]	0.005
Junior high school	−0.30	0.10	9.44	0.74 [0.61–0.90]	0.002
Primary school	−0.25	0.09	7.30	0.78 [0.65–0.93]	0.007
Illiterate or semi-illiterate	0^a^			1	

Dietary habit					
Prefer to meat	−0.75	0.38	3.88	0.47 [0.23–1.00]	0.049
Prefer to vegetables	−0.97	0.36	7.22	0.38 [0.19–0.77]	0.007
Balanced diet	−0.94	0.36	6.79	0.39 [0.19–0.79]	0.009
Vegan	0^a^			1	

Osteoporosis					
No	−0.22	0.10	4.40	0.80 [0.65–0.99]	0.036
Yes	0^a^			1	

Chronic obstructive pulmonary disease					
No	−0.45	0.20	5.02	0.64 [0.43–0.95]	0.025
Yes	0^a^			1	

Cerebral infarction					
No	−0.28	0.09	9.57	0.76 [0.63–0.90]	0.002
Yes	0^a^			1	
Diabetes					
No	−0.16	0.08	4.10	0.85 [0.73–0.99]	0.043
Yes	0^a^			1	

Cold limbs					
No	−0.15	0.07	5.14	0.86 [0.76–0.98]	0.023
Yes	0^a^			1	

Lethargy and laziness in speaking and moving					
No	−0.16	0.08	4.27	0.85 [0.73–0.99]	0.039
Yes	0^a^			1	

Weakness of lower limbs					
No	−0.26	0.08	11.36	0.77 [0.66–0.90]	0.001
Yes	0^a^			1	

Dry throat and mouth					
No	−0.14	0.06	4.65	0.87 [0.77–0.99]	0.031
Yes	0^a^			1	

Slow movement					
No	−0.47	0.10	24.21	0.63 [0.52–0.75]	＜0.001
Yes	0^a^			1	

Glazed expression					
No	−0.56	0.23	5.89	0.57 [0.36–0.90]	0.015
Yes	0^a^			1	

^a^Rreference category when the independent variable is a categorical variable.

**Table 6:** Comparison of models with/without TCM symptoms based on deficiency syndrome for the risk prediction of frailty.

Indicators	Frailty cumulative components (“≥1” vs “＜1”)		Frailty cumulative components (“≥2” vs “＜2”)	
	Reference model	New model	Reference model	New model
AUC (95% CI)	0.69 [0.66–0.71]	0.69 [0.67–0.72]	0.79 [0.76–0.83]	0.81 [0.78–0.85]
*P* value (AUC)	…	0.076	…	0.002
NRI events (%)	…	−44.4	…	−14.0
NRI nonevents (%)	…	52.5	…	55.4
NRI total (%)	…	8.14	…	41.4
*P* value (NRI)	…	0.175	…	0.016
IDI events (%)	…	0.0047	…	0.022
IDI nonevents (%)	…	0.0026	…	0.002
IDI total (%)	…	0.0073	…	0.024
*P* value (IDI)	…	0.056	…	0.041

Baseline model: age, education level, dietary habits, chronic obstructive pulmonary disease, diabetes, cerebral infarction, osteoporosis. New model: baseline model + selected TCM symptoms based on “five viscera” deficiency syndrome (cold limbs, lethargy and laziness in speaking and moving, weakness of lower limbs, slow movement, dry mouth and throat, glazed expression). AUC, area under the receiver-operating characteristic curve; CI, confidence interval; event, incident-specific frailty cumulative components; NRI, continuous net reclassification improvement; IDI, integrated discrimination improvement.

## Data Availability

The data sets generated and analyzed during the current study are not publicly available due to the confidentiality of the data—which is an important component of Shanghai's three-year action plan (2018–2020) for further accelerating the development of traditional Chinese medicine: ZY (2018-2020)-CCCX-4004—but are available from the corresponding author on reasonable request.
